# Short Course in the Microbiome

**DOI:** 10.5772/61257

**Published:** 2015-07-27

**Authors:** Kimberly Falana, Rob Knight, Camilia R. Martin, Romina Goldszmid, K. Leigh Greathouse, Joanne Gere, Howard Young, Winston Patrick Kuo

**Affiliations:** 1 BioPharma Research Council, Tinton Falls, NJ, USA; 2 Department of Pediatrics, Computer Science and Engineering, University of California San Diego, San Diego, CA, USA; 3 Beth Israel Deaconess Medical Center, Harvard Medical School, USA; 4 Laboratory of Experimental Immunology Cancer and Inflammation Program, National Cancer Institute, NIH, USA; 5 Laboratory of Human Carcinogenesis, National Cancer Institute, NIH, USA; 6 Laboratory of Experimental Immunology, National Cancer Institute, NIH, USA; 7 IES Diagnostics, Inc., Cambridge, MA, USA

**Keywords:** microbiome, bacteria, gut, cancer, therapy, antibiotics, prenatal, personalized medicine, short course

## Abstract

Over the past decade, it has become evident that the microbiome is an important environmental factor that affects many physiological processes, such as cell proliferation and differentiation, behaviour, immune function and metabolism. More importantly, it may contribute to a wide variety of diseases, including cancer, inflammatory diseases, metabolic diseases and responses to pathogens. We expect that international, integrative and interdisciplinary translational research teams, along with the emergence of FDA-approved platforms, will set the framework for microbiome-based therapeutics and diagnostics. We recognize that the microbiome ecosystem offers new promise for personalized/precision medicine and targeted treatment for a variety of diseases.

The short course was held as a four-session webinar series in April 2015, taught by pioneers and experts in the microbiome ecosystem, covering a broad range of topics from the healthy microbiome to the effects of an altered microbiome from neonates to adults and the long term effects as it is related to disease, from asthma to cancer. We have learned to appreciate how beneficial our microbes are in breaking down our food, fighting off infections and nurturing our immune system, and this information provides us with ideas as to how we can manipulate our microbiome to prevent certain diseases. However, given the variety of applications, there are scientific challenges, though there are very promising areas in reference to the clinical benefits of understanding more about our microbiome, whether in our gut or on our skin: the outlook is bright. A summary of the short course is presented as a meeting dispatch.

## 1. Introduction

The human microbiome is an array of microorganisms, commonly referred to as the microbiota, which resides in our body and on our skin. Our body is home to about 100 trillion bacteria/microbial cells (in and on our body) and two million microbial genes, but each of us only has about 40 trillion human cells and approximately 20, 000 human genes. Bacterial cells alone outnumber our own by a factor of 20. Therefore, understanding the microbial side and their interactions with us (the host) is of critical importance for our understanding of human biology, the variety of diseases as mentioned, susceptibility to infectious and chronic disease, and even behaviour and drug responses.

The short course on the *Microbiome* organized by the BioPharma Research Council and supported by InTech Open Access Publisher was held as a four-session webinar series in April 2015. The goal of the short course was to provide, at an introductory and in some cases a deeper glance, an exchange between researchers from academia in the current stage of the microbiome in clinical and translational research and, eventually, clinical practice. The microbiome topics included a systems biology approach to understanding our microbiome, a spatially explicit map, how a mother's biota affect physiology, and responses to certain stresses in babies to the role of the microbiome in cancer therapy.

## 2. Session 1: April 9, 2015

### 2.1 The Systems Biology Microbiome Approach by Rob Knight, PhD

The first session was presented by Rob Knight, who provided a systems biology approach/overview to understanding the microbiome. He introduced the current diversity measures used to study the microbiome including alpha diversity, beta diversity, phylogenetic diversity, and taxonomy diversity. For the past decade, Dr. Knight's laboratory focused on developing computational methods/tools for mapping the microbiome data. He discussed the UniFrac method that exploits evolutionary relationships to compare different communities by using sequences to build phylogenetic trees, and then uses the trees to cluster the samples [1, 2].

Dr. Knight highlighted in his talk that the key is to identify connections between microbes and different conditions which we never thought of as being involved, including links between obesity and colon cancer, rheumatoid arthritis and (in mouse models) even things like autism, depression and multiple sclerosis, and finding out which of these conditions microbes cause — and which we can either predict or modify with improved knowledge about the microbial world. As an example of the potential utility of measuring microbial diversity, Dr. Knight described research from his group in which they sequenced gut microbiomes and revealed whether or not someone was obese with an accuracy greater than 90%, apparently more accurate than DNA sequencing [3].

Dr. Knight further explained that faecal microbial samples are a good representation of the gut microbiome and are easy to collect. He presented figures displaying faecal microbial samples from a wide selection of animals clustered together based on a similar diet, gut type and lineage using the UniFrac method [4, 5].

Dr. Knight also explained how researchers are able to distinguish the cause-effect relationship between the microbiome and many related diseases and conditions using Koch's Postulates. As an example, he described research performed using gnotobiotics and model organisms. Through these experiments, scientists discovered that transmissible gut microbiota can determine food intake [6, 7].

Dr. Knight described how, in a Malawian twin study, children with kwashiorkor, or malnutrition, had different microbes to their healthy twins despite having an identical diet. Upon treatment with ready-to-use therapeutic foods, the malnourished twins recovered while their microbial gut population changed, but these effects were only short-term. The microbes from both children in three twin pairs were transferred to germ-free organisms to see if they lost or gained weight. In two cases, those that received microbes from malnourished children lost weight, indicating a causal relationship.

Dr. Knight described a new comprehensive approach involving culturing, gnotobiotics and metagenomics that can help in scientifically determining the impact of our microbiome. In this method, he discussed the methods in which one will isolate the many strains of microbes from a single faecal sample and grow them separately, then mixing them to see if a specific disease phenotype emerges from the mixture. This technique can be utilized to disprove any hypotheses as to whether the cause of the disease phenotype is a virus, cytokine or metabolite as opposed to microbiota [8].

## 3. Session 2: April 16, 2015

### 3.1 Part I: “Roundup of the Microbiome News” by Winston Patrick Kuo, DDS, DMSc, MS

Dr. Kuo gave an insightful overview of recent and exciting discoveries in microbiome research. These discoveries included studies on the relationship of the microbiome to the immune system, obesity, cancer, mental health, and drug metabolism. He discussed how the microbiome affects us directly from birth (whether via traditional or C-section) until we get old, and most of these microbes come from the mother's skin, birth canal and gut. He highlighted in a recent publication how a microbe called *Bifidobacterium* has potentially beneficial effects for babies, as they are among the first microbes to show up in a baby's intestinal tract after birth. Studies suggest a particular type of *Bifidobacteria* can prevent infections and help establish the newborn's immune system. A single gene in the mother called *FUT2* controls the behaviour of *Bifidobacterium*, and this gene works through breast milk [9].

Dr. Kuo mentions how we carry up to 2 kg of microbes in our gut, and two thirds of our gut microbiome is unique to each individual. But the question is: How could your gut microbiota be influencing your health and risk of disease? He describes how the microbes in our gut play an important role in digestion: although sometimes the stomach and small intestine are unable to digest certain foods the gut microbes assist in ensuring we get the nutrients we need. For example, the gut bacteria help in the production of vitamins B and K that play a major role in immune function.

Dr. Kuo highlighted the role of the gut microbiota and an individual's risk of obesity and other metabolic conditions. He discussed the research conducted at Cornell and King's College where they identified a certain strain of bacteria - *Christensenellaceae minuta* - that was more common in people with a low body weight, and that the presence of this particular strain is highly influenced by genes. He mentioned the study was confirmed by introducing this bacteria to the guts of mice, which then caused the animals to gain less weight, indicating the bacteria may reduce or prevent obesity [10, 11].

Dr. Kuo highlighted studies where the gut bacteria were linked to cancers, where researchers discovered specific bacteria in the intestines, *Lactobacillus johnsonii,* that may play a role in the development of lymphoma [12]. He discussed a study conducted by UK researchers that found that a common gut bacteria called *Helicobacter pylori* may cause stomach cancer and duodenal ulcers by deactivating a part of the immune system involved in regulating inflammation. Late last year, investigators from Mount Sinai associated a specific combination of gut bacteria with the development of colorectal cancer [13][Bibr bibr14-61257][Bibr bibr15-61257]–[16].

Dr. Kuo's next topic was the relationship between the microbiome and mental health. Can gut microbes alter the metabolites associated with communication between the gut and the brain, which interferes with brain function? He discussed how bacteria has been shown to play a role in producing neurotransmitters, such as norepinephrine, serotonin, or dopamine, as well as how certain probiotic bacteria can actually modulate the effects of neurotransmitters. He further discussed a microbiome-gut-brain study in germ-free mice, where it was demonstrated that the lack of gut microbes affects sociability, decreases memory, and increases stress responses. The study showed how specific strains of *Lactobacillus rhamnosus* modulate stress, and this effect appears to be mediated through the vagus nerve in mice [17].

Dr. Kuo discussed other recent topics related to stress and anxiety, autoimmune and cardiovascular diseases and drug metabolism. Dr. Kuo concluded his brief overview by stating how we need a better understanding of microbiome and drug interactions so that someday this will allow us to devise strategies to improve drug efficacy and reduce side effects. Not too far ahead, we can start manipulating and explore potential therapeutic uses for the microbiome.

## 4. Session 2: April 16, 2015

### 4.1 Part II: “The Establishment of the Microbiome in Newborn Infants: Challenges and New Opportunities” by Camilia Martin, MD, MS

Dr. Camilia Martin discussed the influences of the early establishment of the microbiome in the maternal-foetal environment and ex-utero determinants. Dr. Martin discussed recent studies that have challenged the dogma that the Maternal-Foetal unit is sterile; however the placenta has been determined to harbour its own unique microbiome, and analysis of the first passage of stool in the infant reveals microorganisms [18, 19]. In addition, she mentioned the analysis of tracheal aspirates after early postnatal intubation reveals organisms, presumably from amniotic fluid swallowed when the infant was in utero [20].

Dr. Martin highlighted how the principle perinatal and postnatal determinants of microbial colonization patterns in the newborn include delivery mode, diet, hospitalization, and medications. Of these factors, a vaginal delivery versus Caesarean section and breast milk versus formula lead to a more favourable microbiome profile that contains known commensal organisms and fewer organisms considered to be pathogenic. In the preterm infant, the microbial pattern is dominated by pathogenic rather than commensal organisms due to factors unique to critically ill populations such as exposure to indigenous hospital organisms and to medications that are known to alter the enteric flora, including antibiotics and H2 blockers [21].

Dr. Martin went on to discuss the medical consequences of an altered microbiome where the establishment of an intestinal microbiome is critical for immune ontogeny and ongoing development of the intestinal tract. She observed how the microbiome profile of a preterm infant appears to have significant health consequences. Unfavourable profiles have been linked to an increased risk of necrotizing enterocolitis, lung disease, and sepsis [20, 22], [23]. The microbiome patterns of older infants and children have also been linked to atopic disease (allergies, asthma), type I diabetes mellitus, celiac disease and obesity [24].

Martin also mentioned how an increasing awareness of the influence of the microbiome on health and risk of disease has already begun to change perinatal maternal and neonatal medical practices. There is a concerted effort to decrease the national Caesarean section rate, increase maternal-infant skin-to-skin colonization after birth, promote early and continued exposure to breast milk, and reduce antibiotic and other medication exposures.

Dr. Martin went ahead to discuss strategies to protect and restore microbial diversity. She emphasized the need to optimize the influence of the microbiome on health, which is critical to protect and restore microbial diversity. For the preterm infant, protecting microbial diversity would entail an understanding of how current medical practices alter the microbiome and a re-evaluation of the implementation of these practices. Restoration of microbial diversity may include promoting dietary strategies that are known to optimize the intestinal microbiome, minimizing the use of medications known to disturb the microbial balance, delivering probiotics (although this remains controversial due to limited well-designed studies), and changing medical practices to those that attempt to emulate natural patterns of colonization, such as strategies to expose the infant to vaginal flora even if delivered by Caesarean section [25].

Dr. Martin concluded by discussing the challenges in bridging the gap between the microbiome and personalized medicine. She mentioned how patterns in the intestinal microbiome have been linked to specific diseases in various populations, overlap is often observed between cases and controls in many of the measures used to define the microbiome. As a result, it is difficult to clinically apply these observations on an individual basis. In parallel, an individual's microbiome profile can be distinctly unique from other individuals such that it serves as a fingerprint to that person's identity. Thus, the precise nature of the influence of the microbiome in an individual's health can be difficult to determine, and one's microbiome-host relationship can be quite distinct from another's. Dr. Martin concluded that non-invasive omics strategies have the potential to increase our understanding of the unique microbial-host interactions bridging the path to both personalized medicine and applied populational health [26].

## 5. Session 3: April 23, 2015

### 5.1 Spatially Explicit Maps by Rob Knight, PhD

In this session, Dr. Knight gave examples of how computational methods can be utilized to map microbiome genomic data. As an example, Dr. Knight presented a map of several microbiome clusters representing oral, vaginal, skin and faecal microbiomes from adults in the same cultural community. Through the sequence of maps, he demonstrated how the faecal microbiome of an infant begins with a composition identical to their mother's vaginal or skin microbiome, and after 2.5 years ends with a composition similar to that of an adult faecal microbiome [27].

As another example, Dr. Knight discussed a study involving spatially explicit maps that illustrated the distribution and diversity of the bacteria on kitchen surfaces. Bacterial samples from four kitchens were tested and averaged. The resulting maps demonstrated relatively high abundances of *Campylobacter* on the stove exhaust fans, *Salmonella* on stoves and sinks, *Clostridium* on the cabinets and *E. coli* on the refrigerator draws. The researchers also differentiated bacteria based on particular sources including skin, produce and faucet water. In relatively high abundance, bacteria derived from the skin were found on trashcans, produce bacteria was found on the stove and counter, and faucet water bacteria was found on faucets. This study emphasizes implications for bacterial survivability, growth and transmission within the kitchen [28].

Dr. Knight believes there is a better way to illustrate the data from such studies than is currently available, and suggests a more spatially explicit approach. Researchers in his lab were able to map the microbial communities of one's face on a map of their face and correlated the different colours to each area of the face based on the composition/make-up of its microbiome.

Dr. Knight highlighted how another emerging and intriguing area of research is establishing and validating the relationships between the metabolome and the microbiome. He discussed how researchers have created microbial and metabolite maps of the whole human body using mass spectroscopy data: by mapping the metabolites and microbiomes onto the human body, researchers can hypothesize which microbes are performing the biotransformations that use or produce those metabolites.

Dr. Knight mentioned that infection by microbial pathogens, such as *MRSA* or *Clostridium difficile,* is highest in a hospital environment. The ultimate goal of the Human Microbiome Project is to reduce the nosocomial infection rate of hospitals through improved disinfection measures and in order to create and support such measures; several factors were studied to determine if they had significant influence on the microbial community and rate of microbial succession within the hospital. These factors included human demographics, physical conditions such as temperature and humidity, building materials, patient microbiota, duration of patient occupancy, patient room and nurse station usage, and the composition and diversity of an existing microbial community derived from previous occupants. In total, 84 different variables were considered. The results will be published in the near future.

## 6. Session 4: April 30, 2015

### 6.1 Part I: Microbiome and Cancer Therapy by Romina Goldszmid, PhD

Dr. Romina Goldszmid discussed how mammals live in partnership with a rich commensal microbiota on their bodies' epithelial surfaces. This partnership is critical for tissue formation, metabolism and the development and function of the innate and adaptive resistance. The microbiota are also closely linked to cancer development both locally (e.g., colorectal carcinoma) and at distant sites (mammary carcinoma, lymphoma). She discussed how recent studies demonstrated that disruption of the commensal gut microbiota impairs the response of subcutaneous cancers to CpG ODN-immunotherapy and platinum chemotherapy, and in both cases innate myeloid cells are responsible for the impaired response, albeit through distinct mechanisms [29]. The failure to respond to immunotherapy was due to the inability of monocyte-derived cells in the tumour microenvironment to produce pro-inflammatory cytokines (e.g., TNF and IL-12) in response to CpG and the subsequent necrosis needed to induce tumour regression.

Dr. Goldszmid further discussed how the composition of the faecal microbiota was distinct among mice displaying high and low TNF responses to CpG treatment, and several bacterial species were found to either positively or negatively correlate with the TNF response. The impaired response to platinum chemotherapy correlated with a lack of an early genotoxic effect of the drug and reduced ROS production by tumour infiltrating myeloid cells. These data point to a role of microbiota in priming tumour-associated myeloid cells to respond to immuno- and chemotherapy.

She highlights how the gut microbiota can also exert an adjuvant effect. For example, certain chemotherapeutics (e.g., cyclophosphamide) or the total body irradiation-conditioning regime performed prior to adoptive T cell transfer therapies cause damage of the gut mucosa allowing bacteria translocation into the draining lymph nodes and increased levels of bacterial products in circulation [30]. The translocated bacteria and their products induce activation of antigen-presenting cells and subsequently the priming of T cells needed for an effective anti-tumour response [31]. Together, these findings suggest that the composition of the commensal microbiota modulates the response to cancer therapy, thus providing new targets and possibilities for therapeutic intervention [32].

## 7. Session 4: April 30, 2015

### 7.1 Part II: Microbiome and Cancer Therapy by K. Leigh Greathouse, PhD, MPH, MS, RD

Dr. Leigh Greathouse discussed that lung cancer is the leading cancer diagnosis worldwide (1.8 million/year) and a major health and financial burden to our healthcare system (US$ 12.1 billion/year). It has a mortality rate higher than that of the top three cancers combined. Epidemiological evidence suggests that alterations in microbial communities due to repeated antibiotic exposure are associated with increased lung cancer risk. The microbiome consists of bacteria, archaea, fungi, eukaryotes and viruses which outnumber host cells ten to one and host genes >100 times.

She elaborated that several bacteria are associated with chronic inflammation and a subsequent increased risk of lung and colon cancer, including *Mycobacterium tuberculosis* (lung cancer) and *Fusobacterium nucleatum* (colon cancer), a bacterium commonly isolated from inflammatory bowel disease patients and a risk factor for colon cancer. The more virulent strains of *F. nucleatum* affect colon cancer progression and increase tumour multiplicity by various mechanisms including favouring the infiltration of tumour-promoting myeloid cells to create a pro-inflammatory environment. Colorectal carcinomas associated with a high abundance of faecal *F. nucleatum* were found to have the highest number of somatic mutations, suggesting that these mutations create a pathogen-friendly environment. Furthermore, the loss of p53 in enterocytes impairs the epithelial barrier and allows infiltration of bacteria, resulting in NF-κB signalling, which was required for tumour progression.

Dr. Greathouse emphasized that the microbiome of lung cancer is largely unknown. Exposure to cigarette smoke reduces epithelial barrier function and increases susceptibility to infections. We hypothesized that somatic mutations together with cigarette smoke create a dysbiotic microbiota that is associated with lung carcinogenesis. To explore this hypothesis, we sequenced 16S rRNA in tissue from lung cancer cases and controls, and lung cancer samples in the Cancer Genome Atlas (TCGA) as validation. Lung cancer cases could be classified by the relative abundance of two taxa, Variovorax and Streptococcus, with an increase in Variovorax abundance in tumours as compared to non-tumour adjacent lung tissue. A group of taxa were significantly associated with squamous cell carcinoma (SCC), of which Acidovorax spp. were enriched in smokers. Further, we observed that these taxa, including Acidovorax, exhibit higher abundance among the subset of SCC cases with TP53 mutations. Therefore, SCC-associated taxa are enriched in tumours with TP53 mutations, establishing a microbiome-gene interaction in lung cancer tissue.

## 8. Conclusion

Based on the session presentations, it was evident that the microbiome translational research is quickly evolving and that it continues to advance in all facets of science. Overall, the short course fulfilled its goal of providing a balanced forum of relevant content from academic researchers. The webinar presentations are available on the BioPharma Research Council website (http://www.biopharmaresearchcouncil.org). Due to the interest level in this space, the BioPharma Research Council and the NCI will co-sponsor a one day event at the National Cancer Institute on September 24, 2015, entitled, “Altering the Microbiome: Can it Impact Health?” The purpose of the meeting is to discuss how the host microbiome can be altered and whether such approaches can result in a positive impact on the host.
